# Contrasting Trends of Smoking Cessation Status: Insights From the Stages of Change Theory Using Repeat Data From the Global Adult Tobacco Survey, Thailand (2009 and 2011) and Turkey (2008 and 2012)

**DOI:** 10.5888/pcd14.160376

**Published:** 2017-06-01

**Authors:** Lazarous Mbulo, Komanduri S. Murty, PhD, Muhammad Jami Husain, PhD, Rizwan Bashir, MSC, Glenda Blutcher-Nelson, BSc, Sarunya Benjakul, PhD, Mondha Kengganpanich, PhD, Toker Erguder, MD, PhD, Bekir Keskinkilic, MD, Sertac Polat, MD, Dhirendra N. Sinha, MD, PhD, Krishna Palipudi, PhD, Indu B. Ahluwalia, PhD, on behalf of the Global Adult Tobacco Survey (GATS) Collaborative Group

**Affiliations:** 1 Global Tobacco Control Branch, Office on Smoking and Health, National Center for Chronic Disease Prevention and Health Promotion, Centers for Disease Control and Prevention, Atlanta, Georgia; 2 Department of Behavioral Sciences, Fort Valley State University, Fort Valley, Georgia; 3 Department of Health Education and Behavioral Sciences, Faculty of Public Health, Mahidol University, Bangkok, Thailand; 4 World Health Organization Country Office, Ankara, Turkey; 5 Ministry of Health, Ankara, Turkey; 6 World Health Organization, South-East Asia Regional Office, New Delhi, India

## Abstract

**Objective:**

The World Health Organization recommends that smokers be offered help to quit. A better understanding of smokers’ interest in and commitment to quitting could guide tobacco control efforts. We assessed temporal differences in stages of change toward quitting among smokers in Thailand and Turkey.

**Methods:**

Two waves (independent samples) of data from the Global Adult Tobacco Survey, a national household survey of adults aged 15 years or older, were assessed for Thailand (2009 and 2011) and Turkey (2008 and 2012). Current smokers were categorized into 3 stages of change based on their cessation status: precontemplation, contemplation, and preparation. Relative change in the proportion of smokers in each stage between waves 1 and 2 was computed for each country.

**Results:**

Between waves, overall current tobacco smoking did not change in Thailand (23.7% to 24.0%) but declined in Turkey (31.2% to 27.1%; *P* < .001). Between 2009 and 2011, precontemplation increased among smokers in Thailand (76.1% to 85.4%; *P* < .001), whereas contemplation (17.6% to 12.0%; *P* < .001) and preparation (6.3% to 2.6%; *P* < .001) declined. Between 2008 and 2012, there were declines in precontemplation among smokers in Turkey (72.2% to 64.6%; *P* < .001), whereas there were increases in contemplation (21.2% to 26.9%; *P* = .008) and no significant change in preparation (6.5% to 8.5%; *P* = .097).

**Conclusion:**

Nearly two-thirds of smokers in Turkey and more than two-thirds in Thailand were in the precontemplation stage during the last survey wave assessed. The proportion of smokers in the preparation stage increased in Turkey but declined in Thailand. Identifying stages of cessation helps guide population-based targeted interventions to support smokers at varying stages of change toward quitting.

## Introduction

Tobacco cessation is associated with many health benefits (1). Accordingly, the World Health Organization (WHO) Framework Convention on Tobacco Control (WHO FCTC) guideline for implementation of Article 14 (2) recognizes the importance of cessation and recommends strengthening or creating a sustainable infrastructure for barrier-free access to quitting resources. One of the WHO MPOWER strategies for effective implementation of the FCTC is to *offer help to quit tobacco use*; thus, member countries are obligated to incorporate tobacco-dependence treatment into national tobacco control programs and health care systems (2). WHO FCTC is an international treaty that presents a blueprint for governments to reduce both supply and demand for tobacco (2). The WHO MPOWER package is a set of 6 proven tobacco control measures to assist country level implementation of FCTC (2).

The implementation of the *offer help to quit tobacco use* strategy warrants a clear understanding of smoking prevalence in general and, more specifically, the cessation stage of smokers. Based on the Transtheoretical Model of stages and processes of change (3), cessation stages are defined as stages a smoker goes through to quit successfully. Understanding these cessation stages can guide efforts to develop and implement tobacco cessation and tobacco dependence treatment programs. For example, smokers who are not thinking of quitting might benefit from interventions to motivate them to attempt to quit smoking, whereas those wanting to quit now may need help finding quitting resources (eg, access to approved medications or behavioral counseling) to successfully quit. Examining cessation stages also allows for the evaluation of tobacco control and prevention programs by characterizing the shifts within cessation stages among smokers.

The Stages of Change, or the Transtheoretical Model, was developed to examine the changes in addictive behaviors (3). The theory was first applied to smoking behavior to validate the model and assess the relationship between stages of change and smoking cessation (3). Tobacco users are categorized into a continuum of 5 stages: precontemplation, contemplation, preparation, action, and maintenance (3). As an integrative biophysiological model, Stages of Change assumes smokers go through the sequence of these stages to quit successfully (3): 1) not at all thinking about quitting (precontemplation), 2) thinking about quitting (contemplation), 3) making preparations to quit (preparation), 4) taking action to quit (action), and 5) quitting and remaining tobacco-free (maintenance). The model can also be used to classify smokers into various cessation stages at both individual and aggregate population levels.

Population-based cessation support is thought to be greatly enhanced through policy and legislative measures that are geared to support the development of a national cessation infrastructure and smoke-free environment (4). To date, since signing and ratifying WHO FCTC in 2004, Thailand and Turkey have implemented most of the WHO MPOWER measures (5) that support cessation efforts among smokers (5). Although Thailand and Turkey are in different regions of the world, both countries have undergone similar significant demographic and democratic processes (6,7) that are important to tobacco control. In both Thailand (8) and Turkey (9), strengthening national tobacco control have been significant policy and legislative processes. Therefore, we examine changes in stages of cessation among smokers in these 2 countries over time, both for the overall population and by demographic characteristics. These results may assist countries with evaluating the effects of their respective tobacco control efforts for cessation and provide data for targeted interventions among smokers at varying stages of change on the cessation continuum.

## Methods

### Data Source

The Global Adult Tobacco Survey (GATS) is a standardized, national household survey of adults aged 15 years or older. The study used 2 waves of GATS data for both Thailand and Turkey. A multistage cluster sampling design was used for both waves of GATS in each country under study to achieve nationally representative samples (10–13). Details of GATS methods, including sampling design and data quality assurance, are available in the GATS manual (14), as well as in individual country GATS reports (10–13).Thailand conducted GATS in 2009 (N = 20,566) and 2011 (N = 20,606), whereas Turkey conducted GATS in 2008 (N = 9,030) and 2012 (N = 9,851). The response rate for Thailand was 94.2% in 2009 and 96.3% in 2011, and the response rate for Turkey was 90.9% in 2008 and 90.1% in 2012.

### Measures

Current tobacco smokers (current smokers) were identified by responses to the question, “Do you currently smoke tobacco on a daily basis, less than daily basis, or not at all?” Respondents who indicated smoking tobacco on a “daily basis” or “less than daily basis” were classified as current smokers.

On the basis of current smoking behavior, duration of smoking, past-year quit attempts, and future cessation intentions, we characterized smokers into 3 mutually exclusive stages of precontemplation, contemplation, and preparation (15–18). Identification of smokers in the action and maintenance stages was considered beyond the scope of the study partly because of the cross-sectional nature of the surveillance data.

A past-year quit attempt was measured by the question, “During the past 12 months, have you tried to stop smoking?” Among those who made a quit attempt (ie, responded yes), the duration of a quit attempt was measured by the question, “Thinking of the last time you tried to quit, how long did you stop smoking?” The response categories were number of months, weeks, days, or “less than 1 day (24 hours).” Information about current smokers’ future intentions to quit was assessed with the question, “Which of the following best describes your thinking about quitting smoking?” The response options included 1) “quit within the next month”; 2) “quit within the next 12 months”; 3) “quit someday, but not next 12 months”; and 4) “not interested in quitting.”

The precontemplation stage included current smokers not thinking of quitting within the next 12 months. Other studies have used the time frame of 6 months for intention to quit (19); however, GATS asked the question using a time frame of 12 months. The contemplation stage included smokers not thinking of quitting within the next month, but considering quitting within the next 12 months. The definition of the preparation stage was similar to that of other studies and included smokers who were thinking of quitting smoking in the next month, as well as those who attempted to quit for at least 24 hours during the previous 12 months (15,18,19).

### Demographic characteristics and analysis

Respondents’ characteristics assessed included the following: sex (male or female); age (15–24 y, 25–44 y, 45–64 y, ≥65 y); residence (urban or rural); education (no formal education/less than primary, completed primary/less than secondary, completed secondary/completed high school, completed college/university or above); wealth index (lowest, low, middle, high, highest); and survey year (ie, year the survey was conducted). Wealth index was used as a proxy measure for socioeconomic status and was constructed using a principal component analysis based on information about household ownership of assets (eg, electricity, flushing toilet, fixed telephone, cellular telephone, television, radio, refrigerator, car, moped/scooter/motorcycle, washing machine). For Thailand and Turkey, a single wealth index was developed by dividing the respondents into quintiles ranging from 1 (lowest) to 5 (highest) (20).

The analysis included only current smokers; former smokers were excluded. Final analytical sample sizes for Thailand were n = 4,901 in 2009 and n = 4,209 in 2011. Final analytical sample sizes for Turkey were n = 2,701 in 2008 and n = 2,412 in 2012.

Estimates were calculated for current smokers among all respondents, as well as the proportion of smokers in each of the assessed stages of change (ie, precontemplation, contemplation, and preparation), by wave and demographic characteristic. In addition, for each country, relative changes were calculated for each stage between wave 1 and wave 2, both overall and by demographic characteristic; a z-test was used to assess significance at *P* < .05. A multinomial logistic regression analysis was performed modeling the odds of stage of change (category outcome) as a function of covariates (age, sex, education, residence, wealth index, and survey year); the Wald *F*-test was used to evaluate significance between groups at *P* < .05. All data were weighted to account for the complex survey design in each country and to yield nationally representative estimates. Data were analyzed with SPSS version 18.0 (IBM Corporation).

## Results

### Thailand

The overall smoking prevalence in Thailand remained unchanged, from 23.7% in 2009 to 24.0% in 2011. Smoking prevalence remained unchanged among both male and female respondents (Figure). More comparisons are presented in the country report (11).

**Figure:**
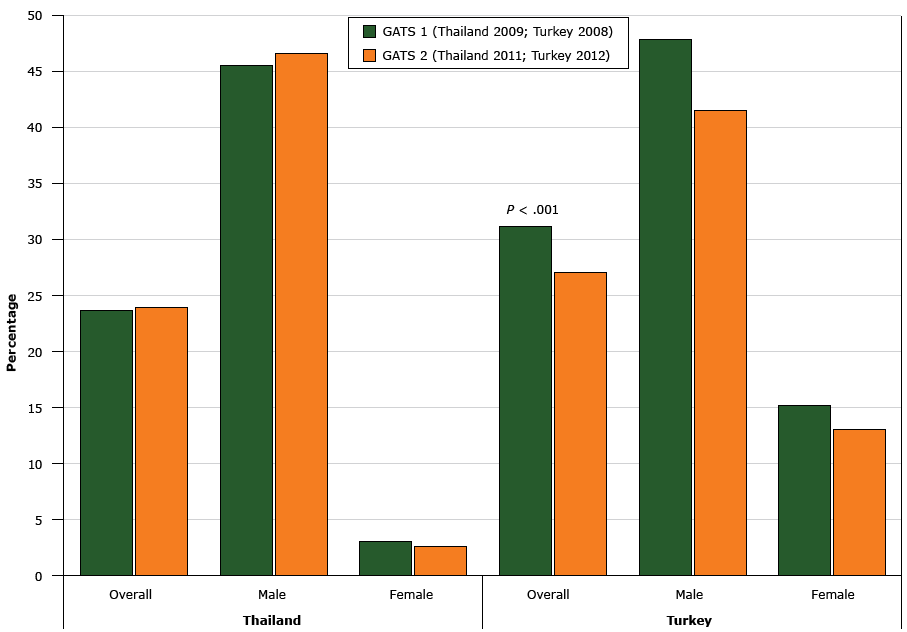
Tobacco Smoking Prevalence in Thailand (2009 and 2011) and Turkey (2008 and 2012), Global Adult Tobacco Survey (GATS). ^a^ The decline in prevalence in Turkey from 2008 to 2012 was significant (*P* < .001). **Table:** 

Sex	GATS 1		GATS 2	
	Thailand, 2009 %	Turkey, 2008 %	Thailand, 2011 %	Turkey, 2012 %
Overall	23.7	31.2^a^	24.0	27.1^a^
Male	45.6	47.9	46.6	41.5
Female	3.1	15.2	2.6	13.1

Table 1 shows the distribution of smokers in Thailand by demographic characteristics across the 3 stages of change assessed and by wave. At wave 1 in Thailand, 76.1% of smokers were in precontemplation, 17.6% in contemplation, and 6.3% in preparation. In wave 2, 85.4% were in precontemplation, 12.0% in contemplation, and 2.6% in preparation (*P* < .001 for all).

**Table 1:** Distribution of Tobacco Smokers^a^ in Thailand According to Stages of Change Cessation Stage, Global Adult Tobacco Survey, Thailand, 2009 and 2011

Demographic Characteristics	n^b^	Precontemplation	Contemplation	Preparation	n^b^	Precontemplation	Contemplation	Preparation
	2009 % (95% CI)				2011 % (95% CI)			
**Overall**	4,901	76.1 (73.8–78.1)	17.6 (15.8–19.6)	6.3 (5.3–7.5)	4,290	85.4 (83.4–87.2)	12.0 (10.3–13.9)	2.6 (2.0–3.5)
**Sex**								
Male	4,469	76.3 (74.0–78.4)	17.6 (15.7–19.6)	6.1 (5.1–7.4)	3,892	85.7 (83.5–87.6)	11.8 (10.1–13.8)	2.5 (1.9–3.4)
Female	432	72.9 (65.6–79.2)	18.5 (13.4–25.1)	8.6 (5.4–13.3)	398	81.0 (75.5–85.4)	14.7 (10.6–20.1)	4.3 (2.5–7.3)
**Age, y**								
15–24	455	75.6 (69.0–81.1)	16.4 (11.9–22.1)	8.0 (5.1–12.3)	418	86.0 (79.9–90.5)	12.0 (7.7–18.1)	2.0 (0.8–4.7)
25–44	2,091	75.6 (72.6–78.5)	17.9 (15.6–20.5)	6.5 (5.1–8.2)	1,662	84.6 (81.5–87.2)	12.1 (9.8–14.9)	3.3 (2.3–4.7)
45–64	1,779	77.4 (74.5–80.1)	17.3 (15.0–20.0)	5.2 (4.1–6.7)	1,686	86.8 (84.3–88.8)	11.3 (9.3–13.6)	2.0 (1.4–2.8)
≥65	576	74.5 (69.0–79.2)	19.8 (15.6–24.8)	5.7 (3.8–8.7)	524	82.8 (78.1–86.7)	14.2 (10.6–18.9)	3.0 (1.7–5.2)
**Residence**								
Urban	2,695	74.2 (71.7–76.5)	18.1 (16.3–20.1)	7.7 (6.4–9.3)	2,288	81.4 (79.0–83.6)	14.4 (12.4–16.7)	4.1 (3.1–5.5)
Rural	2,206	76.8 (73.8–79.5)	17.5 (15.1–20.1)	5.8 (4.5–7.4)	2,002	87.1 (84.3–89.4)	10.9 (8.8–13.5)	2.0 (1.3–3.1)
**Education**								
No formal education/less than primary	1,883	77.6 (74.6–80.2)	17.4 (15.1–20.1)	5.0 (3.8–6.5)	1,608	87.8 (85.4–89.9)	10.2 (8.3–12.4)	2.0 (1.3–3.1)
Completed primary/less than secondary	1,068	78.6 (74.5–82.2)	16.7 (13.6–20.5)	4.6 (3.3–6.5)	920	85.1 (80.3–88.9)	11.8 (8.4–16.4)	3.0 (1.7–5.3)
Completed secondary/completed high school	1,690	73.1 (69.4–76.6)	18.3 (15.4–21.6)	8.5 (6.6–11.0)	1,314	84.2 (81.0–87.0)	13.5 (10.9–16.4)	2.3 (1.5–3.7)
Completed college/university or above	252	72.3 (62.9–80.1)	19.5 (13.6–27.2)	8.2 (3.6–17.6)	447	81.9 (75.2–87.1)	13.1 (8.4–19.8)	5.0 (3.0–8.2)
**Wealth Index**								
Lowest	1,280	77.9 (74.4–81.1)	15.0 (12.5–18.0)	7.0 (5.2–9.3)	1,184	86.6 (83.3–89.4)	11.2 (8.7–14.3)	2.2 (1.4–3.5)
Low	1,124	78.6 (74.3–82.4)	16.7 (13.3–20.9)	4.7 (3.4–6.4)	995	86.6 (82.5–89.8)	10.1 (7.4–13.7)	3.3 (1.9–5.6)
Middle	1,269	74.9 (71.2–78.2)	18.8 (15.9–22.3)	6.3 (4.5–8.6)	849	85.2 (80.5–89.0)	13.3 (9.7–18.0)	1.4 (0.5–3.7)
High	646	73.2 (68.1–77.8)	19.3 (15.6–23.6)	7.5 (4.7–11.5)	803	83.3 (79.2–86.8)	13.8 (10.5–17.9)	2.9 (1.8–4.5)
Highest	582	71.8 (66.2–76.8)	21.0 (16.1–26.9)	7.2 (4.6–11.0)	459	84.5 (79.9–88.2)	11.3 (8.3–15.2)	4.2 (2.3–7.4)

Abbreviation: CI, confidence interval.

a Current tobacco smokers (ie, adults who smoke tobacco on a daily basis or less than daily basis).

b N is the sample size for the smoker population only.

In Thailand, the distribution of smokers by stages of change did not vary significantly across demographic groups in either wave 1 or 2. In 2009, the proportion of smokers in the precontemplation stage was 76.1% overall, ranging from 71.8% among the highest wealth index to 78.6% among those who completed primary or less than secondary education and those with a low wealth index. The proportion of smokers in the contemplation stage was 17.6% overall, ranging from 16.4% among those aged 15 to 24 years to 21.0% among those with the highest wealth index. The proportion of smokers in the preparation stage was 6.3% overall, ranging from 4.6% among those who completed primary or less than secondary education to 8.6% among female respondents.

In 2011, the proportion of smokers in the precontemplation stage was 85.4% overall, ranging from 81.0% among female respondents to 87.8% among those who completed no formal education or less than primary education; the proportion of smokers in the contemplation stage was 12.0% overall, ranging from 10.1% among those with a low wealth index, to 14.7% among female respondents. The proportion of smokers in the preparation stage was 2.6% overall and ranged from 1.4% among those in the middle wealth index to 5.0% among those who completed college or university.

Table 2 presents the relative percentage change in Thailand for each stage across demographic categories between wave 1 and wave 2. In Thailand, a significant (*P* < .001) overall relative increase of 12.3% occurred among smokers in precontemplation during the 2-year period (2009 to 2011). The relative change in precontemplation varied from 8.3% among those who completed primary or less than secondary school to 17.7% among those in the highest wealth index. A significant relative change was also observed across all demographic groups, with the exception of respondents who completed college or university or above. For the contemplation stage, there was a significant decline overall and among all demographic categories except for female respondents and those aged 15 to 24 years. Similarly, a significant decline occurred among respondents in the preparation stage, both overall and among all demographic subgroups, with the exception of 3 groups: those who completed primary education or less than secondary education, those who completed college or university or above, and those in the low wealth index.

**Table 2:** Relative Change in Distribution of Tobacco Smokers^a^ According to Stages of Change Cessation Stage, Global Adult Tobacco Survey, Thailand (2009–2011) and Turkey (2008–2012)

Demographic Characteristics	Precontemplation	Contemplation	Preparation	Precontemplation	Contemplation	Preparation
	Thailand, 2009–2011, Relative % Change (95% CI)			Turkey, 2008–2012, Relative % Change (95% CI)		
**Overall**	12.3 (8.2 to 16.4)	−32.2 (−45.8 to −19.8)	−58.4 (−72.1 to −44.6)	−10.6 (−15.4 to −5.9)	27.0 (10.7 to 43.3)	29.9 (−2.0 to 61.8)
**Sex**						
Male	12.3 (8.1 to 16.5)	−32.9 (−45.7 to −20.0)	−58.9 (−73.3 to −44.5)	−9.9 (−15.1 to −4.6)	26.1 (8.3 to 43.9)	25.0 (−10.6 to 60.5)
Female	11.0 (−1.4 to 23.5)	−20.5 (−56.2 to 15.3)	−49.5 (−85.0 to −14.1)	−13.0 (−21.6 to −4.5)	29.7 (−1.2 to 60.6)	44.5 (−12.6 to 101.7)
**Age, y**						
15–24	13.8 (2.3 to 25.3)	−27.0 (−65.5 to 11.5)	−75.0 (−99.3 to −50.8)	0.8 (−10.7 to 12.4)	−11.6 (−44.3 to 21.0)	26.5 (−43.0 to 96.0)
25–44	11.9 (6.1 to 17.6)	−32.4 (−49.4 to −15.4)	−49.0 (−71.4 to −26.6)	−15.8 (−21.7 to −9.8)	43.6 (20.4 to 66.8)	45.7 (−0.5 to 91.9)
45–64	12.1 (7.1 to 17.0)	−35.0 (−50.5 to −19.5)	−62.3 (−78.9 to −45.7)	−6.9 (−15.2 to 1.4)	19.1 (−7.8 to 46.0)	7.9 (−40.0 to 55.9)
≥65	11.2 (1.7 to 20.8)	−28.1 (−54.8 to −1.4)	−48.6 (−85.1 to −12.0)	−7.2 (−24.6 to 10.3)	28.5 (−49.8 to 106.8)	0 (−93.6 to 93.7)
**Residence**						
Urban	9.8 (5.1 to 14.6)	−20.4 (−34.7 to −6.0)	−46.6 (−65.3 to −27.9)	−12.0 (−17.7 to −6.2)	30.4 (9.5 to 51.3)	42.0 (−1.7 to 85.8)
Rural	13.4 (8.0 to 18.7)	−37.3 (−53.4 to −21.3)	−65.2 (−83.1 to −47.3)	−6.8 (−14.1 to 0.5)	19.4 (−2.3 to 41.1)	1.9 (−34.0 to 37.7)
**Education**						
No formal education/less than primary	13.2 (8.2 to 18.2)	−41.4 (−55.8 to −27.0)	−60.0 (−80.1 to −40.0)	−9.7 (−23.2 to 3.8)	28.6 (−34.2 to 91.5)	52.0 (−84.1 to 188.1)
Completed primary/less than secondary	8.3 (0.7 to 15.9)	−29.3 (−57.0 to −1.7)	−34.8 (−78.3 to 8.6)	−10.1 (−16.7 to −3.4)	20.8 (−1.7 to 43.4)	46.4 (−1.4 to 94.3)
Completed secondary/completed high school	15.1 (8.1 to 22.1)	−26.6 (−46.0 to −7.1)	−72.5 (−87.3 to −57.8)	−10.2 (−17.3 to −3.0)	29.2 (4.5 to 54.0)	13.1 (−32.1 to 58.4)
Completed college/university or above	13.2 (−2.6 to 29.0)	−32.9 (−70.0 to 4.2)	−38.6 (−96.8 to 19.6)	−11.6 (−24.8 to 1.7)	32.7 (−15.1 to 80.5)	4.1 (−59.6 to 67.8)
**Wealth Index**						
Lowest	11.1 (4.9 to 17.3)	−25.7 (−48.8 to −2.5)	−68.5 (−85.9 to −51.1)	0.2 (−11.4 to 11.7)	4.1 (−36.1 to 44.3)	−11.4 (−63.8 to 41.1)
Low	10.1 (2.8 to 17.4)	−39.4 (−62.5 to −16.2)	−29.2 (−73.1 to 14.7)	−10.7 (−20.0 to −1.3)	28.2 (−6.3 to 62.8)	34.1 (−39.2 to 107.4)
Middle	13.8 (6.0 to 21.6)	−29.2 (−54.2 to −4.3)	−77.1 (−100 to −54.1)	−15.5 (−23.9 to −7.2)	51.0 (9.8 to 92.2)	34.7 (−26.3 to 95.7)
High	13.8 (4.7 to 22.9)	−28.6 (−52.6 to −4.6)	−61.3 (−86.0 to −36.6)	−9.8 (−17.9 to −1.7)	14.8 (−10.8 to 40.4)	60.9 (−15.0 to 136.8)
Highest	17.7 (7.2 to 28.2)	−46.1 (−67.4 to −24.7)	−42.1 (−84.4 to 0.2)	−12.2 (−23.5 to −1.0)	29.0 (−5.6 to 63.7)	18.9 (−42.1 to 80.0)

a Current tobacco smokers (ie, adults who smoke tobacco on a daily basis or less than daily basis).

Multinomial logistic regression analysis for Thailand showed that only the effects of the survey year (Wald *F* = 33.49, *P* < .001) were significant. Age (Wald *F* =1.65, *P* = .19), sex (Wald *F* = 3.0, *P* = .05), education level (Wald *F* = 0.59, *P* = .74), residence (Wald *F* = 1.62, *P* = .20), and wealth index (Wald *F* = 0.69, *P* = .70) were not significant in differentiating the Stages of Change categories (data not shown in tables).

### Turkey

Overall smoking prevalence in Turkey declined significantly, from 31.2% in 2008 to 27.1% in 2012 (*P* < .001). Declines in smoking prevalence were further noted among both male and female respondents (Figure). Detailed comparisons across demographic characteristics are presented in the country report (13). Among current smokers, wave 1 showed that 72.2% of smokers were in the precontemplation stage, 21.2% were in contemplation, and 6.5% were in preparation. Wave 2 showed 64.6% in precontemplation (*P* < .001), 26.9% in contemplation (*P* = .008), and 8.5% in preparation (*P* = .097).

Demographic differences were observed among smokers by stage of change between waves 1 and 2 (Table 3). In 2008, the proportion of smokers in the different stages ranged as follows: the precontemplation stage, from 68.4% (highest wealth index) to 77.3% (no formal education or less than primary education); the contemplation stage, from 18.3% (no formal education or less than primary education) to 23.7% (highest wealth index); and the preparation stage, from 4.4% (no formal education or less than primary education) to 8.3% (college education or university or above).

**Table 3:** Distribution of Tobacco Smokers^a^ According to Stages of Change Cessation Stage, Global Adult Tobacco Survey, Turkey, 2008 and 2012

Demographic Characteristics	n^b^	Precontemplation	Contemplation	Preparation	n^b^	Precontemplation	Contemplation	Preparation
	2008, % (95% CI)				2012, % (95% CI)			
**Overall**	2,701	72.2 (69.8–74.6)	21.2 (19.3–23.2)	6.5 (5.5–7.8)	2,412	64.6 (61.8–67.3)	26.9 (24.6–29.4)	8.5 (7.2–10.0)
**Sex**								
Male	2,036	72.4 (69.7–74.9)	21.2 (19.1–23.4)	6.5 (5.2–7.9)	1,782	65.2 (62.1–68.2)	26.7 (24.1–29.4)	8.1 (6.6–9.8)
Female	665	71.8 (67.4–75.9)	21.4 (17.8–25.5)	6.8 (5.0–9.2)	630	62.5 (57.4–67.2)	27.7 (23.6–32.3)	9.8 (7.6–12.6)
**Age, y**								
15–24	303	70.2 (64.3–75.4)	22.3 (17.4–28.0)	7.6 (5.1–11.1)	247	70.7 (64.5–76.3)	19.7 (14.7–25.9)	9.6 (6.4–14.1)
25–44	1,511	73.6 (70.6–76.4)	20.4 (18.0–23.1)	5.9 (4.7–7.5)	1,343	62.0 (58.3–65.6)	29.3 (26.3–32.5)	8.7 (7.0–10.7)
45–64	764	70.3 (66.0–74.4)	22.4 (19.0–26.4)	7.2 (5.2–10.0)	694	65.5 (61.0–69.7)	26.7 (22.8–31.1)	7.8 (5.8–10.4)
≥65	123	75.2 (64.8–83.3)	18.9 (11.6–29.4)	5.9 (2.9–11.8)	128	69.8 (59.0–78.7)	24.3 (16.1–35.1)	5.9 (3.2–10.8)
**Residence**								
Urban	1,522	73.4 (70.4–76.2)	20.4 (18.2–22.9)	6.1 (4.8–7.7)	1,401	64.7 (61.2–68.0)	26.7 (23.8–29.8)	8.7 (7.1–10.6)
Rural	1,179	68.9 (64.9–72.6)	23.4 (20.1–26.9)	7.7 (5.9–10.1)	1,011	64.2 (60.6–67.7)	27.9 (24.9–31.1)	7.9 (6.2–10.0)
**Education**								
No formal education/less than primary	240	77.3 (70.2–83.1)	18.3 (12.9–25.4)	4.4 (2.4–8.0)	154	69.8 (60.5–77.7)	23.5 (16.3–32.8)	6.7 (3.4–12.7)
Completed primary/less than secondary	1,396	72.7 (69.3–75.8)	21.0 (18.3–23.9)	6.3 (4.9–8.1)	1,137	65.4 (61.4–69.1)	25.3 (22.1–28.8)	9.3 (7.5–11.5)
Completed secondary/completed high school	834	71.4 (67.7–74.8)	21.7 (18.8–25.0)	6.9 (5.1–9.2)	835	64.1 (60.0–68.0)	28.1 (24.6–31.8)	7.8 (5.9–10.2)
Completed college/university or above	231	68.5 (60.8–75.2)	23.2 (17.0–30.9)	8.3 (5.3–12.8)	286	60.5 (53.9–66.8)	30.8 (24.9–37.4)	8.7 (5.6–13.2)
**Wealth Index**								
Lowest	464	71.8 (65.9–76.9)	20.1 (15.2–26.0)	8.2 (5.4–12.3)	350	71.9 (65.3–77.6)	20.9 (15.6–27.3)	7.3 (4.7–11.0)
Low	543	73.4 (68.4–77.8)	21.4 (17.2–26.3)	5.2 (3.5–7.8)	498	65.5 (59.9–70.8)	27.5 (23.1–32.3)	7.0 (4.8–10.2)
Middle	580	75.1 (70.2–79.5)	18.7 (14.8–23.2)	6.2 (4.4–8.6)	623	63.5 (58.4–68.2)	28.2 (23.9–32.8)	8.4 (6.1–11.3)
High	672	71.7 (67.5–75.6)	22.1 (18.7–26.0)	6.2 (4.3–8.8)	606	64.7 (60.0–69.1)	25.4 (21.8–29.4)	9.9 (7.2–13.4)
Highest	442	68.4 (62.6–73.7)	23.7 (19.4–28.7)	7.8 (5.4–11.1)	335	60.1 (54.0–65.9)	30.6 (25.3–36.5)	9.3 (6.4–13.4)

a Current tobacco smokers (ie, adults who smoke tobacco on a daily basis or less than daily basis).

b N is the sample size for the smoker population only.

In 2012, the proportion of smokers in the different stages ranged as follows: the precontemplation stage, from 60.1% (highest wealth index) to 71.9% (lowest wealth index); the contemplation stage, from 19.7% (aged 15 to 24 years) to 30.8% (college or university education or above); and the preparation stage, from 5.9% (aged ≥65 years) to 9.9% (high wealth index).

Table 2 presents the relative percentage change in Turkey for each stage across demographic categories between wave 1 and wave 2. A significant relative decline of 10.6% occurred in the proportion of smokers in the precontemplation stage overall. Among subgroups, significant declines were also observed among male respondents; female respondents; those aged 25 to 44 years; those who completed primary education or less than secondary education, secondary education or high school, or college or university or above; and those in low wealth index to the highest wealth index. In contrast, a significant overall increase occurred in the proportion of smokers in the contemplation stage; among subgroups, significant increases were also observed among male respondents; female respondents; those aged 25 to 44 years; those who lived in both rural and urban areas; those who completed primary or less than secondary education or secondary education or high school; and those in the middle wealth index. An overall increase was observed in the proportion of smokers in the preparation stage, but the change was not significant (*P* = .097).

 Multinomial logistic regression results for Turkey showed that only the effects of survey year (Wald *F* = 9.73, *P* < .001) were significant. Age (Wald *F* = 0.05, *P* = .95), sex (Wald *F* = 0.41, *P* = .67), education level (Wald *F* = 0.44, *P* = .85), residence (Wald *F* = 1.34, *P* = .26), and wealth index (Wald *F* = 1.42, *P* = .18) were not significant in differentiating the Stages of Change categories (data not shown in tables).

## Discussion

Our findings show significant changes in cessation stages among smokers in both Thailand and Turkey; however, the direction of change varied between countries. In Thailand, there was an increase in precontemplation (not thinking about quitting) with a concurrent decline in contemplation (thinking about quitting) and preparation (making plans to quit). However, in Turkey, there was a decrease in precontemplation with a concurrent increase in contemplation with no change in preparation. In addition, multinomial logistic regression model results showed a significant relationship between year and stage of change. However, the model suggests that stages of change among smokers are not influenced by age, sex, education level, residence, and wealth index. Taken together, the results in this study suggest that both countries could benefit from continued adoption of population-level interventions that are proven to motivate smokers to quit and provision of tailored support to smokers in the contemplation and preparation stages.

The reasons for the differences in stages of change across years and between countries observed in this study could be multifactorial, including differences in how each country implements their tobacco control policies. Since signing the WHO FCTC in 2004, Thailand has incrementally introduced significant policy measures, including pictorial warnings on cigarette packs and a tax increase, that could have motivated more smokers into thinking about quitting (8,21). However, it is possible that inadequate cessation infrastructure or poor enforcement of tobacco control policies in Thailand could have resulted in reduced interest in quitting among interested smokers at advanced stages of change (22). The results may also demonstrate relapse and recycle through the stages among smokers as they attempt to modify or quit their addictive behavior (23).

Conversely, Turkey has made significant amendments to the Prevention and Control of Hazards of Tobacco Products law in 2008 (9). Several of these measures — including prohibitions on smoking in enclosed public places, prohibitions on tobacco advertising, and increases in tobacco product taxes — may have helped denormalize smoking and fostered interest in quitting among smokers (9).

Most smokers in both Thailand and Turkey were in the precontemplation stage in both waves, findings that are consistent with those of other population-based studies (18,24,25). Therefore, these results present potential directions for interventions that target most smokers. However, shifts within stages of change, including relapses to an earlier stage (23), may suggest a more comprehensive approach to cessation. Adopting population-based interventions targeting precontemplators and contemplators to move them to the next stage could also be accompanied by strategies that target preparation and action stages. For example, each country could adopt comprehensive smoke-free policies in public places, raise tobacco taxes, and use effective media campaigns (26), targeting precontemplators and contemplators while providing effective behavioral and pharmacological treatment of tobacco dependence. These efforts could be achieved with the use of effective low-cost strategies that support tobacco cessation among those who wish to quit even when medication is not available (27,28). This strategy could help both countries to increase quit attempts and successful quitting that are important to reducing diseases and deaths due to tobacco use.

The findings of this study have limitations. First, the study used self-reported data, which is subject to misreporting. Second, the cross-sectional nature of the data limits our ability to draw any causal conclusions about changes in smoking behavior from any specific events or tobacco control activities. Third, variations within countries and times of surveys limit the comparability of the results. Finally, the Stages of Change model only considers a smoker who plans to quit in the next month, unless he or she has made a recent quit attempt, to be in the preparation stage (29); therefore, smokers with a plan to quit at a time longer than a month away were not captured.

In conclusion, nearly two-thirds of smokers in Turkey and more than two-thirds of smokers in Thailand were in the precontemplation stage of cessation. The proportion of smokers strongly committed to quitting (ie, in the preparation stage) remained the same in recent years in Turkey but declined in Thailand. These findings suggest that tailored tobacco control interventions, coupled with continuous surveillance of tobacco use and changes in cessation stages, could guide tobacco control policy and practice and, more specifically, help tobacco control efforts with FCTC Article 14, which aim to create a sustainable infrastructure to motivate quit attempts and ensure wide access to cessation support for tobacco users.

## References

[1] The health benefits of smoking cessation. A report of the Surgeon General. Washington (DC): US Department of Health and Human Services; 1990. 32255575

[2] World Health Organization. Guidelines for implementation of Article 14 of the WHO Framework Convention on Tobacco Control. Fourth session in November 2010, the Conference of the Parties (COP) 2010. http://www.who.int/fctc/Guidelines.pdf?ua=1. Accessed October 2016.

[3] Prochaska JO , DiClemente CC . Stages and processes of self-change of smoking: toward an integrative model of change. J Consult Clin Psychol 1983;51(3):390–5. 10.1037/0022-006X.51.3.390 6863699

[4] Global status report on noncommunicable diseases 2010: description of the global burden of NCDs, their risk factors and determinants. Geneva (CH): World Health Organization; 2011.

[5] WHO report on the global tobacco epidemic, 2013. Enforcing bans on tobacco advertising, promotion and sponsorship. Geneva (CH): World Health Organization; 2013.

[6] McCargo D , Zarakol A . Turkey and Thailand unlikely twins. J Democracy 2012;23(3):1–10. 10.1353/jod.2012.0055

[7] Zarakol A . Revisiting second image reversed: lessons from Turkey and Thailand. Int Stud Q 2013;57(1):150–62. 10.1111/isqu.12038

[8] Chantornvong S , McCargo D . Political economy of tobacco control in Thailand. Tob Control 2001;10(1):48–54. 10.1136/tc.10.1.48 11226361 PMC1763992

[9] Bilir N , Özcebe H , Ergüder T , Mauer-Stender K. Tobacco control in Turkey: story of commitment and leadership. Copenhagen (DK): WHO-Euro; 2012.

[10] World Health Organization, Regional Office for South East Asia. Global Adult Tobacco Survey: Thailand report. Nonthaburi (TH): Thailand Ministry of Health; 2009. http://www.who.int/tobacco/surveillance/thailand_gats_report_2009.pdf. Accessed October 2016.

[11] World Health Organization, Regional Office for South East Asia. Global Adult Tobacco Survey: Thailand report. Nonthaburi (TH): Thailand Ministry of Health; 2011. http://www.searo.who.int/tobacco/surveillance/Global_Adult_Tobacco_Survey_Thailand_Report_2011.pdf. Accessed October 2016.

[12] Turkey, Ministry of Health. Global Adult Tobacco Survey: Turkey 2010. Ankara (TR): Ministry of Health, 2010. http://www.who.int/tobacco/surveillance/en_tfi_gats_turkey_2009.pdf?ua=1. Accessed October 2016.

[13] Turkey, Ministry of Health. Global Adult Tobacco Survey: Turkey 2012. Ankara (TR): Ministry of Health; 2014. http://www.who.int/tobacco/surveillance/survey/gats/report_tur_2012.pdf?ua=1. Accessed October 2016.

[14] Global Adult Tobacco Survey Collaborative Group. Global adult tobacco surveys (GATS): sample design manual. Atlanta (GA): Centers for Disease Control and Prevention; 2010.

[15] Etter J-F , Perneger TV , Ronchi A . Distributions of smokers by stage: international comparison and association with smoking prevalence. Prev Med 1997;26(4):580–5. 10.1006/pmed.1997.0179 9245682

[16] DiClemente CC , Prochaska JO , Fairhurst SK , Velicer WF , Velasquez MM , Rossi JS . The process of smoking cessation: an analysis of precontemplation, contemplation, and preparation stages of change. J Consult Clin Psychol 1991;59(2):295–304. 10.1037/0022-006X.59.2.295 2030191

[17] Etter J-F , Sutton S . Assessing ‘stage of change’ in current and former smokers. Addiction 2002;97(9):1171–82. 10.1046/j.1360-0443.2002.00198.x 12199833

[18] Velicer WF , Prochaska JO , Fava JL , Rossi JS , Redding CA , Laforge RG , Using the Transtheoretical Model for population-based approaches to health promotion and disease prevention. Homeost Health Dis 2000;40:174–95.

[19] DiClemente CC , Prochaska JO , Fairhurst SK , Velicer WF , Velasquez MM , Rossi JS . The process of smoking cessation: an analysis of precontemplation, contemplation, and preparation stages of change. J Consult Clin Psychol 1991;59(2):295–304. 10.1037/0022-006X.59.2.295 2030191

[20] Palipudi KM , Gupta PC , Sinha DN , Andes LJ , Asma S , McAfee T ; GATS Collaborative Group. Social determinants of health and tobacco use in thirteen low and middle income countries: evidence from Global Adult Tobacco Survey. PLoS One 2012;7(3):e33466. 10.1371/journal.pone.0033466 22438937 PMC3306395

[21] World Health Organization. Joint national capacity assessment on the implementation of effective tobacco control policies in Thailand. Geneva (CH): World Health Organization; 2011.

[22] Vathesatogkit P . Benefits that Thailand tobacco control law and program bring to the country. Tobacco and Health. World Health Organization: 1996–2002, 2008.

[23] Prochaska JO , DiClemente CC , Norcross JC . In search of how people change. Applications to addictive behaviors. Am Psychol 1992;47(9):1102–14. 10.1037/0003-066X.47.9.1102 1329589

[24] Mbulo L , Palipudi KM , Nelson-Blutcher G , Murty KS , Asma S ; Global Adult Tobacco Survey Collaborative Group. The process of cessation among current tobacco smokers: a cross-sectional data analysis from 21 countries, Global Adult Tobacco Survey, 2009–2013. Prev Chronic Dis 2015;12:150146. 10.5888/pcd12.150146 26378897 PMC4576423

[25] Piper ME , Kenford S , Fiore MC , Baker TB . Smoking cessation and quality of life: changes in life satisfaction over 3 years following a quit attempt. Ann Behav Med 2012;43(2):262–70. 10.1007/s12160-011-9329-2 22160762 PMC3298628

[26] Institute of Medicine. Ending the tobacco problem. A blueprint for the nation. Washington (DC): National Academies Press; 2007.

[27] Lando HA . Future research needs and capacity building. Presentation at the WHO meeting on Global Policy for Smoking Cessation hosted by the Ministry of Health of the Russian Federation. Moscow; June 14–15, 2002.

[28] US Department of Health and Human Services. Clinical practice guideline: treating tobacco use and dependence. Rockville (MD): Agency for Healthcare Research and Quality, 2000.

[29] Sutton S . A critical review of the Transtheoretical Model applied to smoking cessation. In: Norman P, Abraham C, Conner M (editors). Understanding and changing health behaviour: from health beliefs to self-regulation (pp. 207–25). Amsterdam (NL): Harwood Academic Publishers; 2000.

